# Perinatal Depression of Exposed Maternal Women in the COVID-19 Pandemic in Wuhan, China

**DOI:** 10.3389/fpsyt.2020.551812

**Published:** 2020-12-16

**Authors:** Guoqiang Sun, Qi Wang, Ying Lin, Ruyan Li, Lijun Yang, Xian Liu, Min Peng, Hongyan Wang, Xuewen Yang, Wei Ren, Hui Yang, Yao Cheng

**Affiliations:** ^1^Obstetrics Department, Maternal and Child Health Hospital of Hubei Province, Huazhong University of Science and Technology, Wuhan, China; ^2^Department of Epidemiology and Biostatistics, School of Public Health, Tongji Medical College, Huazhong University of Science and Technology, Wuhan, China

**Keywords:** COVID-19, EPDS, prenatal, postnatal, depression

## Abstract

**Objective:** This study aims to investigate perinatal depression in women who gave birth during the COVID-19 pandemic in Wuhan, and to evaluate the effect of the pandemic on perinatal depression prevalence.

**Methods:** A cross-sectional investigation was conducted into women hospitalized for delivery in Hubei Maternity and Child Healthcare Hospital from December 31, 2019 to March 22, 2020, a period which encompasses the entire time frame of the COVID-19 pandemic in Wuhan. The Edinburgh Postnatal Depression Scale (EPDS) was adopted to evaluate perinatal depression status. A Chi-square test and logistic regression model were utilized for data analysis.

**Results:** A total of 2,883 participants were included, 33.71% of whom were found to suffer from depressive symptoms. In detail, 27.02%, 5.24%, and 1.46% were designated as having mild, moderate, and severe depressive symptoms, respectively. The perinatal depression prevalence increased as the COVID-19 pandemic worsened. Compared to the period from December 31, 2019 to January 12, 2020, perinatal depression risk significantly decreased within the 3 weeks of March 2–22, 2020 (1st week: OR = 0.39, 95% CI: 0.20, 0.78; 2nd week: OR = 0.35, 95% CI: 0.17, 0.73; and 3rd week: OR = 0.48, 95% CI: 0.25, 0.94); and the postnatal depression risk significantly rose within the four weeks of January 27-February 23, 2020 (1st week: OR = 1.78, 95% CI: 1.18, 2.68; 2nd week: OR = 2.03, 95% CI: 1.35, 3.04; 3rd week: OR = 1.48, 95% CI: 1.02, 2.14; and 4th week: OR = 1.73, 95% CI: 1.20, 2.48).

**Conclusion:** The dynamic change of perinatal depression was associated with the progression of the COVID-19 pandemic among new mothers who were exposed to the pandemic. An elevated risk of postnatal depression was also observed during the COVID-19 pandemic.

## Introduction

In December 2019, a novel virus, officially named SARS-CoV-2, caused an outbreak of pneumonia in Wuhan. The disease was named coronavirus disease 2019 (COVID-19) by the WHO. The newly identified SARS-CoV-2 virus was one of high transmissibility (1). The COVID-19 pandemic has since spread across China and around the world. On January 30, 2020, the WHO declared the virus a public health emergency of international concern, and then a worldwide pandemic on March 12, 2020 (2). The ongoing pandemic has posed a great threat to the physical and mental health of affected individuals.

Chinese governments have taken extensive and efficient actions to control the pandemic. Many provinces and regions in China implemented highest-level public health measures in response to the emergency. The *Guidance Manual on the Prevention and Control of Novel Coronavirus-Infection Pneumonia in the Community* issued by the national government on January 28, 2020, suggested traffic restrictions, home quarantine, and other social distancing measures (3). Besides, to avoid cross-infection opportunities in hospitals, designated hospitals and medical centers were established to exclusively treat the SARS-CoV-2 infection.

Global public health emergencies may promote psychological disorders in affected individuals. The severe acute respiratory syndrome (SARS), which caused a worldwide epidemic in 2003, equipped China with valuable lessons on how to control and prevent COVID-19, and has evidenced the importance of public psychological crisis management in the control of major public health events (4). The SARS epidemic was demonstrated to have negative psychological effects on infected patients (5), health professionals (6), students (7), and the general public (8). Scholars have called for attention to be drawn to the psychological disorders related to COVID-19 pandemic exposure (9). To tackle the potential psychological problems of affected individuals, the Chinese Center for Disease Control and Prevention issued the *Guiding Principles for Intervention in Emergency Psychological Crises during the Novel Coronavirus-Infection Pneumonia Epidemic*, and announced that an intervention of psychological crises should be involved in the whole deployment of COVID-19 pandemic prevention and control (10). Moreover, some hospitals and psychologists provided free online courses on psychological crisis treatment for COVID-19 affected individuals (11).

However, there are limited mental health services available for maternal women, as well as evidence for efficient management against the psychological effect of the pandemic. As a vulnerable group affected by high levels of estrogen and progesterone, the upper respiratory tract mucosa of pregnant women thickens causing edema and mild congestion, which are prone to respiratory infection. And SARS-CoV-2 infected pregnant patients were presented to have poor prognoses. Therefore, perinatal mothers deserve priority in psychological health guarantees (12). Maternal depression is one of the most common complications among perinatal women, which may result in devastating life events to mothers, infants, and families (13). Perinatal depression may have negative effects on offspring well-being throughout their whole life (14–16). The mortality rate attributed to perinatal depression-resulted suicide even exceeds that caused by postpartum hemorrhage and pregnancy induced hypertension (17). New mothers need delivery-related medical services in hospitals where they may be confronted with nosocomial SARS-CoV-2 infection, which possibly increases their psychological crisis during the perinatal period. With all this in mind, the perinatal depression status of maternal women is worthy of assessment during the entire COVID-19 pandemic period.

This study aims to investigate the prevalence of perinatal depression in hospitalized maternal women and to evaluate the depression risk in relation to COVID-19 pandemic exposure. The findings of our study will provide helpful experiences for the handling of subsequent large-scale psychological crises among perinatal women.

## Methods

### Data Sources and Study Population

The participants were general healthy pregnant women receiving delivery services at Hubei Maternity and Child Healthcare Hospital in Wuhan, one of the largest cities in central China. The hospital is the largest Class III Grade A maternity center in Hubei Province. In 2018, one out of five newborns in Wuhan were expected to be delivered in this hospital. Their delivery help services mainly provided for women not infected with COVID-19 during the pandemic.

The study conducted included hospitalized individuals with a gestation of 28 weeks and above or ≤7 days after delivery (as of the perinatal period). Our study was approved by the Ethics Committee of the hospital. The cross-sectional study was implemented from December 31, 2019 to March 22, 2020, a period that spanned the whole COVID-19 pandemic in Wuhan. All participants provided oral informed consent before the investigation, in line with the Declaration of Helsinki regarding human participation. The questionnaire survey was mainly completed online using the WeChat-based survey program Questionnaire Star (questionnaire link: https://www.wjx.cn/jq/55187836.aspx), which was wildly used during the COVID-19 pandemic. A total of 5% of the participants completed a paper questionnaire with the exact same items due to the temporary unavailability of their mobile phone in hospital.

### Variable Definitions

#### Exposure Variable

The COVID-19 pandemic was regarded as an exposure variable. The daily reported number of confirmed COVID-19 patients was key to the psychological responses of the exposed new mothers, manifesting perinatal depression during the entire epidemic period. Sequential events occurred with the updated measures against the pandemic, including: 1) the first reported patients with unknown pneumonia on December 31, 2019; 2) newly reported patients with an onset of January 16, 2020 on January 18, 2020; 3) the official announcement of the human-to-human transmission on January 20, 2020; 4) the beginning of inner-city traffic restrictions and home quarantine on January 23, 2020; 5) centralized treatment and quarantine strategies to the confirmed patients, suspected patients, patients with a fever symptom, and close contacts; and 6) the first reported zero number of new COVID-19 patients up to March 18, 2020 on March 19, 2020 (18, 19). In response to the concurrent COVID-19 pandemic, maternal women may suffer a different degree of perinatal depression throughout several time intervals.

#### Covariates

Covariates included sociodemographic variables such as age (<25, 25–29, 30–34, and > 34), ethnicity (Han and other), education (junior high or below, senior high, and college or above), Hukou (urban and rural), annual family income (<50,000 ¥, 50,000–100,000 ¥, more than 100,000 ¥, and unknown), delivery status (prenatal and postnatal), gravidity (1, 2–3, and ≥4), parity (0–1 and ≥2), gestational age (<37 and ≥37), health-related behaviors like prior history of traumatic delivery experiences (yes and no), sleep quality (good, fair, and poor), smoking (no, yes, and passive smoking), alcohol drinking (no and yes), and exercise (no and yes) during pregnancy.

Otherwise, most mothers lived with their family and family members played an important role in their social interaction. An Adaptation, Partnership, Growth, Affection, and Resolution Scale (APGAR) was used to evaluate the family function of each respondent. The scale was developed by Smilkstein and has been widely used (20, 21). It consists of five dimensions (adaptation, partnership, growth, affection, and resolution) (22). Each item was rated on a scale from 0 (*hardly ever*) to 2 (*almost always*). The APGAR scale was translated into Chinese in this study and was confirmed to be of good internal consistency (Cronbach's α = 0.80). A final summarized score of 0–3, 4–6, and 7–10 indicated poor, fair, and good family functions (23).

#### Dependent Variables

The Edinburgh Postnatal Depression Scale (EPDS) was used for perinatal depression assessment. The EPDS was compiled by Cox et al. (24) and has been proven to have good reliability and validity, and be suitable for depression assessment during pregnancy and postpartum periods. The scale comprises 10 items, each of which is scored on a four-point scale ranging from 0 to 3 by severity. Finally, the summarized score is classified into four grades, with 0–9, 10–16, 17–21, and 22–30 points indicating none, mild, moderate, and severe depression levels, respectively (25). The internal consistency of this scale was good in our study (Cronbach's α = 0.92).

### Statistical Analysis

The statistical analyses were performed using SAS 9.4 for Windows. A Chi-square test was used to analyze differences in categorical variables, including sociodemographic variables, health behavior factors, and the exposure variable. Multiple logistic regression models adjusted for the mentioned covariates were used to evaluate the dependency of perinatal depression risks on the exposures. Results of the multiple logistic regression model were reported as adjusted odds ratios (OR) and corresponding 95% confidence intervals (CI). All analyses were two-sided and a *p*-value of <0.05 was considered statistically significant.

## Results

**Figure 1:**
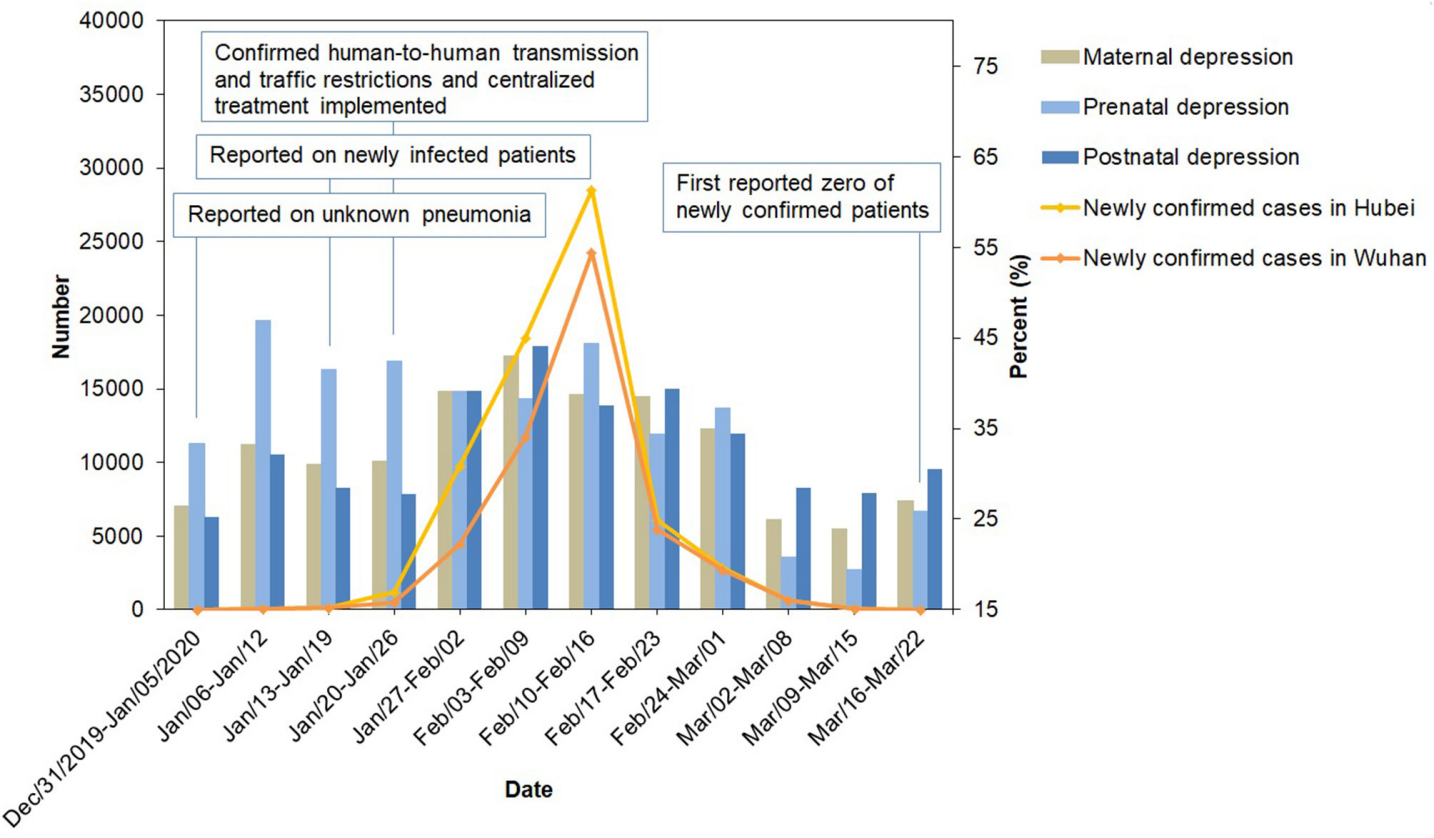
The newly confirmed cases of COVID-19 and the prevalence of perinatal depression in Wuhan from December 31, 2019 to March 22, 2020. Surveillance data of COVID-19 were obtained from the official website of the Health Commission of Hubei Province (http://wjw.hubei.gov.cn/fbjd/dtyw/).

A total of 4,895 inpatients in the hospital were eligible during the study period, and 2,937 of them agreed to participate in the survey. After removing those with incomplete data (*n* = 18) and with prior depression (*n* = 36), 2,883 participants were included in the statistical analysis. Figure 1 shows the number of daily confirmed COVID-19 cases and the perinatal depression prevalence per week during the study period. From the end of 2019 when the first COVID-19 cases emerged in Wuhan, the perinatal depression prevalence continued to increase along with the increasing number of daily reported COVID-19 cases. During the time intervals of January 13–19 and February 3–9, the daily reported number of new cases increased rapidly in Wuhan. Meanwhile, the depression prevalence rose from 30.99% within the week of January 13–19 to 42.98% within the week of February 3–9. The highest prenatal depression value was 46.97% within the week of January 6–12. And the highest postnatal value was 44.15% within the week of February 3–9. As the emergence of SARS-CoV-2 infections slowed down, the perinatal depression prevalence showed a downward trend from 42.98% during February 3–9 to 23.85% during March 9–15.

Table 1 and Supplementary Table 1 show the sociodemographic characteristics of participants stratified by depression levels. In total, 33.71% of all participants were found to have depressive symptoms, 27.02, 5.24, and 1.46% of which were indicative of mild, moderate, and severe depression, respectively (Supplementary Table 1). The majority of participants were aged 25–29 (41.42%), of Han race (97.29%), highly educated (79.01%), urban (64.61%), and had a family income of ≥ 100,000 ¥ (52.03%). The depression prevalence was higher (of marginal significance) in the participants over 34 years of age than those of ≤ 34 years (16.36 vs. 12.82%, *p* = 0.0462).

**Table 1:** Descriptive statistics for the sociodemographic variables of participants.

**Variables**	**No depression N (%)**	**Depression N (%)**	**Total N (%)**	***χ^2^***	***P***
Age				7.9910	0.0462
<25	86 (4.50)	40 (4.12)	126 (4.37)		
25-29	789 (41.29)	405 (41.67)	1,194 (41.42)		
30-34	791 (41.39)	368 (37.86)	1,159 (40.20)		
>34	245 (12.82)	159 (16.36)	404 (14.01)		
Total	1,911 (66.29)	972 (33.71)	2,883 (100.00)		
Ethnicity				0.1006	0.7511
Han	1,857 (97.23)	947 (97.43)	2,804 (97.29)		
Other	53 (2.77)	25 (2.57)	78 (2.71)		
Education					
Junior high or below	136 (7.12)	69 (7.10)	205 (7.11)	0.4932	0.7814
Senior high	259 (13.55)	141 (14.51)	400 (13.87)		
College or more	1,516 (79.33)	762 (78.40)	2,278 (79.01)		
Hukou				0.0008	0.9775
Rural	676 (35.37)	344 (35.43)	1,020 (35.39)		
Urban	1,235 (64.63)	627 (64.57)	1,862 (64.61)		
Family income (¥)				3.2618	0.3530
<50,000	233 (12.19)	127 (13.07)	360 (12.49)		
50,000-100,000	419 (21.93)	232 (23.87)	651 (22.58)		
≥100,000	1,017 (53.22)	483 (49.69)	1,500 (52.03)		
Unclear	242 (12.66)	130 (13.37)	372 (12.90)		
Delivery status				1.4369	0.2306
Prenatal	504 (26.78)	234 (24.68)	738 (26.08)		
Postnatal	1,378 (73.22)	714 (75.32)	2,092 (73.92)		
Gravidity				0.3191	0.5721
1	852 (46.18)	449 (47.26)	1,301 (46.55)		
2-3	799 (43.31)	405 (42.63)	1204 (43.08)		
≥4	194 (10.51)	96 (10.11)	290 (10.38)		
Parity				0.1639	0.6856
0-1	1,225 (66.40)	638 (67.16)	1,863 (66.65)		
≥2	620 (33.60)	312 (32.84)	932 (33.35)		
Gestational age (weeks)				7.5788	0.0059
<37	168 (9.11)	118 (12.45)	286 (10.24)		
≥37	1,676 (90.89)	830 (87.55)	2,506 (89.76)		

*No depression: EPDS score 0-9; depression: EPDS score 10-30*.

Table 2 and Supplementary Table 2 show the health behavior factors stratified by depression levels. Most of the participants (92.47%) reported no previous traumatic delivery experience; and those who experienced a traumatic delivery had a higher depression prevalence (10.36 vs. 6.08%, *p* < 0.0001). Only 52.37% of participants reported a good sleep quality during pregnancy. Participants with a poor or fair sleep quality had significantly higher depression prevalence than those with a good sleep quality. Moreover, most participants did not smoke or drink alcohol during gestation. Compared to those without smoking habits, the participants who smoked either actively or passively had higher depression prevalence (5.37 vs. 2.95%, *p* = 0.0013). Participants who did not exercise during pregnancy had more prevalent depression than those who exercised regularly (44.81 vs. 37.26%, *p* < 0.0001). The majority of participants (90.67%) reported good family functions, and those reporting poor or fair family functions had significantly higher depression prevalence.

**Table 2:** Descriptive statistics for the health behavior factors of participants during pregnancy.

**Variables**	**No depression N (%)**	**Depression N (%)**	**Total N (%)**	***χ^2^***	***P***
Traumatic delivery experience				16.8512	<0.0001
Yes	115 (6.08)	100 (10.36)	215 (7.53)		
No	1,777 (93.92)	865 (89.64)	2,642 (92.47)		
Sleep quality				193.2296	<0.0001
Good	1,156 (60.94)	345 (35.60)	1,501 (52.37)		
Fair	661 (34.84)	495 (51.08)	1,156 (40.33)		
Poor	80 (4.22)	129 (13.31)	209 (7.29)		
Smoking				10.3242	0.0013
No	1,840 (97.05)	916 (94.63)	2,756 (96.23)		
Yes	56 (2.95)	52 (5.37)	108 (3.77)		
Drinking				0.7171	0.3971
No	1,851 (97.63)	937 (97.10)	2,788 (97.45)		
Yes	45 (2.37)	28 (2.90)	73 (2.55)		
Exercise				15.2252	<0.0001
No	706 (37.26)	432 (44.81)	1,138 (39.80)		
Yes	1,189 (62.74)	532 (55.19)	1,721 (60.20)		
Family function				77.5428	<0.0001
Poor	29 (1.52)	24 (2.47)	53 (1.84)		
Fair	73 (3.82)	143 (14.71)	216 (7.49)		
Good	1,809 (94.66)	805 (82.82)	2,614 (90.67)		

*No depression: EPDS score 0-9; depression: EPDS score 10-30*.

Table 3 and Supplementary Table 3 show the dynamic changes of exposures and depression levels of participants. The depression prevalence in the participants was significantly different during several time intervals throughout the COVID-19 pandemic.

**Table 3:** Descriptive statistics for independent variables of participants during the COVID-19 epidemic.

**Variables**	**No depression N (%)**	**Depression N (%)**	**Total N (%)**	***χ^2^***	***P***
Period of COVID-19 epidemic				45.4120	<0.0001
December 31, 2019–January 5, 2020	78 (4.08)	28 (2.88)	106 (3.68)		
January 6–January 12	177 (9.26)	88 (9.05)	265 (9.19)		
January 13–January 19	167 (8.74)	75 (7.72)	242 (8.39)		
January 20–January 26	103 (5.39)	47 (4.84)	150 (5.20)		
January 27–February 2	143 (7.48)	92 (9.47)	235 (8.15)		
February 3–February 9	134 (7.01)	101 (10.39)	235 (8.15)		
February 10–February 16	213 (11.15)	135 (13.89)	348 (12.07)		
February 17–February 23	223 (11.67)	140 (14.40)	363 (12.59)		
February 24–March 1	203 (10.62)	109 (11.21)	312 (10.82)		
March 2–March 8	193 (10.10)	64 (6.58)	257 (8.91)		
March 9–March 15	166 (8.69)	52 (5.35)	218 (7.56)		
March 16–March 22	111 (5.81)	41 (4.22)	152 (5.27)		

*No depression: EPDS score 0-9; depression: EPDS score 10-30*.

Table 4 shows the results of the logistic regression model for the effects of the COVID-19 pandemic on perinatal depression. Compared to the 1st week (December 31, 2019–January 12, 2020) of the pandemic, the prenatal depression risk was significantly decreased during the 3 week period starting from March 2, 2020 (1st week: OR = 0.39, 95% CI: 0.20, 0.78; 2nd week: OR = 0.35, 95% CI: 0.17, 0.73; and 3rd week: OR = 0.48, 95% CI: 0.25, 0.94), whereas the postnatal depression risk significantly rose during the 4 weeks between January 27 and February 23, 2020 (1st week: OR = 1.78, 95% CI: 1.18, 2.68; 2nd week: OR = 2.03, 95% CI: 1.35, 3.04; 3rd week: OR = 1.48, 95% CI: 1.02, 2.14; and 4th week: OR = 1.73, 95% CI: 1.20, 2.48).

**Table 4:** Logistic regression analysis for the effects of independent variables on prenatal depression and postnatal depression.

	**Prenatal depression**			**Postnatal depression**		
**Variables**	**Crude OR (95% CI)**	**Adjusted OR (95% CI)^a^**	**Adjusted OR (95% CI)^b^**	**Crude OR (95% CI)**	**Adjusted OR (95% CI)^a^**	**Adjusted OR (95% CI)^b^**
Period of COVID-19 epidemic (in reference to December 31, 2019–January 5, 2020)						
January 13–January 19	0.84 (0.42, 1.66)	0.89 (0.43, 1.84)	0.75 (0.36, 1.56)	0.93 (0.62, 1.40)	0.92 (0.61, 1.39)	0.98 (0.64, 1.50)
January 20–January 26	0.84 (0.36, 1.95)	1.19 (0.46, 3.08)	0.85 (0.35, 2.08)	0.92 (0.57, 1.48)	0.89 (0.55, 1.43)	0.90 (0.55, 1.48)
January 27–February 2	0.92 (0.43, 1.95)	1.01 (0.46, 2.19)	1.06 (0.48, 2.37)	1.67 (1.13, 2.48)*	1.67 (1.12, 2.47)*	1.78 (1.18, 2.68)**
February 3–February 9	0.86 (0.41, 1.82)	0.94 (0.43, 2.07)	1.02 (0.45, 2.31)	2.05 (1.39, 3.03)***	2.03 (1.37, 3.00)***	2.03 (1.35, 3.04)***
February 10–February 16	1.07 (0.54, 2.11)	1.38 (0.68, 2.82)	1.37 (0.65, 2.89)	1.54 (1.08, 2.19)*	1.53 (1.07, 2.19)*	1.48 (1.02, 2.14)*
February 17–February 23	0.71 (0.35, 1.44)	0.77 (0.36, 1.62)	0.77 (0.36, 1.65)	1.71 (1.21, 2.42)**	1.71 (1.20, 2.42)**	1.73 (1.20, 2.48)**
February 24–March 1	0.80 (0.40, 1.63)	0.86 (0.41, 1.79)	0.85 (0.39, 1.83)	1.40 (0.97, 2.02)	1.41 (0.97, 2.04)	1.42 (0.96, 2.09)
March 2–March 8	0.35 (0.18, 0.66)**	0.36 (0.18, 0.71)**	0.39 (0.20, 0.78)**	1.08 (0.68, 1.70)	1.08 (0.69, 1.71)	1.08 (0.67, 1.73)
March 9–March 15	0.34 (0.17, 0.67)**	0.38 (0.19, 0.79)**	0.35 (0.17, 0.73)**	0.95 (0.58, 1.54)	0.93 (0.57, 1.52)	0.90 (0.54, 1.50)
March 16–March 22	0.44 (0.24, 0.83)*	0.49 (0.25, 0.95)*	0.48 (0.25, 0.94)*	1.24 (0.58, 2.64)	1.18 (0.55, 2.53)	1.27 (0.58, 2.79)

a *Adjusted for sociodemographic variables including age, ethnicity, education, Hukou, family income, delivery status, gravidity, parity, and gestational age*;

b *Adjusted for sociodemographic and health behavior factors including age, ethnicity, education, Hukou, family income, delivery status, gravidity, parity, gestational age, traumatic delivery experience, sleep quality, smoking, drinking, exercise, and family function*.

***p < 0.001, **p < 0.01, *p < 0.05.

## Discussion

In this study, the prevalence rate of perinatal depression was reported among the hospitalized pregnant women exposed to the COVID-19 pandemic in Wuhan. The prevalence of perinatal depression per week ranged from 23.85 to 42.98%. The overall prevalence of perinatal depression was higher in low- and middle-income countries (19–25%), than that in developed countries (7–15%) (26, 27). It should be noticed that a population-based participant enrolment method was adopted in other studies, whereas a hospital-based method was used in our study. Participants enrolled using the different methods possibly differ in general characteristics, and hence differ in perinatal depression risk. Moreover, the participants had just experienced or were about to experience a delivery, which might cause an increased risk of perinatal depression for them.

The prevalence of prenatal depression was at a high level during the COVID-19 pandemic in Wuhan. Okagbue et al. performed a review study of 26 articles, observing that the prevalence of EPDS-evaluated prenatal depression was 23.8% in pregnant women in their third trimester (28). Huang et al. conducted a cross-sectional survey of 320 pregnant women in their second and third trimesters in Wuhan, reporting a depression prevalence of 29.06% (29). Another study reported that prenatal depression prevalence ranged from 22.10 to 31.94% in Shiyan City, Hubei Province (30, 31). The prenatal depression prevalence in this study was higher than Western China (14.2%) and lower than Eastern China (34.9–36.8%) (32–35).

These results are a warning that the COVID-19 pandemic may possibly bring negative mental impacts to pregnant women all over the world. Controlling for sociodemographic and health behavior variables, the risk of prenatal depression decreased during the period of March 2–22, 2020 compared with the counterpart value at the beginning of the pandemic (December 31, 2019–12 January 12, 2020). The obscure and confusing public information about the pandemic during January 6–12, 2020 may have contributed to the high prenatal depression prevalence in this period. The first report of COVID-19 was published on December 31, 2019; however, the official announcement of the human-to-human transmission of COVID-19 did not occur until January 20, 2020. With implementations of public health measures against the pandemic, including isolation, quarantine, social distancing, and community containment, etc., the daily reported number of new onsets continued to decline, which was smaller than 200 per day after March 1, 2020 (18). This achievement in the prevention and control of the pandemic may be a reason for the decreased prenatal depression prevalence during that period. Flowers et al. studied the biopsychosocial effect of influenza pandemics, showing that pregnant women were vulnerable to a sense of pressure concerning influenza pandemics and death from influenza (36). As a negative and stressful life event, the COVID-19 pandemic probably increased mental depression. It was found that negative life events during pregnancy were associated with increased depression in the third trimester (37–39). The previous SARS outbreak which caused an international public health crisis, has been proven to have had adverse effects on people's mental health, especially in the severely affected areas (40, 41). Otherwise, the Wuhan city quarantine measure started on January 23, 2020 may also have increased postnatal psychological pressure.

This study is the first to reveal the prevalence of perinatal depression in individuals exposed to the COVID-19 pandemic. Some limitations of the study should be noted. First, as a cross-sectional study, it was not able to confirm a causal relationship between the COVID-19 pandemic and depression risks. Second, we enrolled generally healthy prenatal and postnatal women without SARS-CoV-2 infection in this study. And all participants were from a single maternity institution located in the seriously affected area (with respect to the number of COVID-19 cases). Prudence is needed to extend the research conclusions to those SARS-CoV-2 infected individuals and other areas. Third, the study was hospital-based, which possibly resulted in the limited representability of participants and hence limited the extensionality of our conclusions. Forth, this study included only the inpatient participants recruited in perinatal periods. The mental health effects of exposures in the first and second trimesters still need to be studied in the COVID-19 pandemic.

During the COVID-19 pandemic, intervention measures against perinatal depression are crucial for the well-being of new mothers and babies. The dynamic change of perinatal depression was associated with COVID-19 pandemic progression among the maternal women studied. An elevated risk of postnatal depression was also observed during the COVID-19 pandemic.

## Data Availability Statement

The raw data supporting the conclusions of this article will be made available by the authors, without undue reservation.

## Ethics Statement

The studies involving human participants were reviewed and approved by the Ethics Committee of Hubei Maternal and Child Health Care Hospital. Written informed consent for participation was not required for this study in accordance with the national legislation and the institutional requirements.

## Author Contributions

GS: design, data collection, and manuscript editing. QW: data collection, data interpretation, and literature search. YC: manuscript draft, design, data collection, and data analysis. YL, RL, LY, and XL: data collection, data interpretation, and manuscript review. MP, HW, and XY: data collection, manuscript review, and visualization. WR and HY: data collection, data validation, and manuscript revision. All of the authors gave final approval of the version to be submitted, and agree to be accountable for all aspects of the work.

## Conflict of Interest

The authors declare that the research was conducted in the absence of any commercial or financial relationships that could be construed as a potential conflict of interest.
