# Supplementary material for: Perinatal Depression of Exposed Maternal Women in the COVID-19 Pandemic in Wuhan, China
Source: Front Psychiatry. 2020 Dec 16;11:551812. doi: 10.3389/fpsyt.2020.551812 (PMC7772463; doi:10.3389/fpsyt.2020.551812)
Supplement: Supplementary file 1 [file Data_Sheet_1.docx]

**Table S1. Descriptive statistics for the sociodemographic variables of participants**.

| **Variables** | **No**  **N (%)** | **Mild**  **N (%)** | **Moderate**  **N (%)** | **Severe**  **N (%)** | ***χ^2^*** | ***P*** |
| --- | --- | --- | --- | --- | --- | --- |
| Age |  |  |  |  | 5.6696 | 0.1288 |
| <25 | 86 (4.50) | 31 (3.98) | 6 (3.97) | 3 (7.14) |  |  |
| 25-29 | 789 (41.29) | 321 (41.21) | 68 (45.03) | 16 (38.10) |  |  |
| 30-34 | 791 (41.39) | 297 (38.13) | 55 (36.42) | 16 (38.10) |  |  |
| >34 | 245 (12.82) | 130 (16.69) | 22 (14.57) | 7 (16.67) |  |  |
| Total | 1911 (66.29) | 779 (27.02) | 151 (5.24) | 42 (1.46) |  |  |
| Ethnicity |  |  |  |  | 0.0537 | 0.8167 |
| Han | 1857 (97.23) | 761 (97.69) | 146 (96.69) | 40 (95.24) |  |  |
| Other | 53 (2.77) | 18 (2.31) | 5 (3.31) | 2 (4.76) |  |  |
| Education |  |  |  |  |  |  |
| Junior high or below | 136 (7.12) | 53 (6.80) | 11 (7.28) | 5 (11.90) | 0.2415 | 0.8863 |
| Senior high | 259 (13.55) | 120 (15.40) | 14 (9.27) | 7 (16.67) |  |  |
| College or more | 1516 (79.33) | 606 (77.79) | 126 (83.44) | 30 (71.43) |  |  |
| Hukou |  |  |  |  | 0.0279 | 0.8674 |
| Rural | 676 (35.37) | 278 (35.73) | 52 (34.44) | 14 (33.33) |  |  |
| Urban | 1235 (64.63) | 500 (64.27) | 99 (65.56) | 28 (66.67) |  |  |
| Family income (¥) |  |  |  |  | 4.3103 | 0.2299 |
| <50,000 | 233 (12.19) | 96 (12.32) | 22 (14.57) | 9 (21.43) |  |  |
| 50,000-10,000 | 419 (21.93) | 187 (24.01) | 33 (21.85) | 12 (28.57) |  |  |
| ≥10,000 | 1017 (53.22) | 388 (49.81) | 78 (51.66) | 17 (40.48) |  |  |
| Unclear | 242 (12.66) | 108 (13.86) | 18 (11.92) | 4 (9.52) |  |  |
| Delivery status |  |  |  |  | 0.6544 | 0.4186 |
| Prenatal | 504 (26.78) | 183 (24.05) | 42 (28.57) | 9 (22.50) |  |  |
| Postnatal | 1378 (73.22) | 578 (75.95) | 105 (71.43) | 31 (77.50) |  |  |
| Gravidity |  |  |  |  | 0.2245 | 0.8938 |
| 1 | 852 (46.18) | 359 (47.30) | 74 (49.66) | 16 (38.10) |  |  |
| 2-3 | 799 (43.31) | 325 (42.82) | 63 (42.28) | 17 (40.48) |  |  |
| ≥4 | 194 (10.51) | 75 (9.88) | 12 (8.05) | 9 (21.43) |  |  |
| Parity |  |  |  |  | 0.1094 | 0.7408 |
| 0-1 | 1225 (66.40) | 509 (67.06) | 102 (68.46) | 27 (64.29) |  |  |
| ≥2 | 620 (33.60) | 250 (32.94) | 47 (31.54) | 15 (35.71) |  |  |
| Gestational age (weeks) |  |  |  |  | 10.3002 | 0.0013 |
| <37 | 168 (9.11) | 87 (11.48) | 25 (16.89) | 6 (14.29) |  |  |
| ≥37 | 1676 (90.89) | 671 (88.52) | 123 (83.11) | 36 (85.71) |  |  |

No: EPDS score 0-9; Mild: EPDS score 10-16; Moderate: EPDS score 17-21; Severe: EPDS score 22-30.

**Table S2. Descriptive statistics for the health behavior factors of participants during pregnancy.**

| **Variables** | **No**  **N (%)** | **Mild**  **N (%)** | **Moderate**  **N (%)** | **Severe**  **N (%)** | ***χ^2^*** | ***P*** |
| --- | --- | --- | --- | --- | --- | --- |
| Traumatic delivery experience | | |  |  | 20.9488 | <0.0001 |
| Yes | 115 (6.08) | 76 (9.84) | 15 (9.93) | 9 (21.43) |  |  |
| No | 1777 (93.92) | 696 (90.16) | 136 (90.07) | 33 (78.57) |  |  |
| Sleep quality |  |  |  |  | 222.1392 | <0.0001 |
| Good | 1156 (60.94) | 300 (38.66) | 37 (24.50) | 8 (19.05) |  |  |
| Fair | 661 (34.84) | 389 (50.13) | 86 (56.95) | 20 (47.62) |  |  |
| Poor | 80 (4.22) | 87 (11.21) | 28 (18.54) | 14 (33.33) |  |  |
| Smoking |  |  |  |  | 13.3572 | 0.0003 |
| No | 1840 (97.05) | 735 (94.84) | 145 (96.03) | 36 (85.71) |  |  |
| Yes | 56 (2.95) | 40 (5.16) | 6 (3.97) | 6 (14.29) |  |  |
| Drinking |  |  |  |  | 1.7666 | 0.1838 |
| No | 1851 (97.63) | 753 (97.41) | 144 (96.00) | 40 (95.24) |  |  |
| Yes | 45 (2.37) | 20 (2.59) | 6 (4.00) | 2 (4.76) |  |  |
| Exercise |  |  |  |  | 18.1506 | <0.0001 |
| No | 706 (37.26) | 333 (43.19) | 79 (52.32) | 20 (47.62) |  |  |
| Yes | 1189 (62.74) | 438 (56.81) | 72 (47.68) | 22 (52.38) |  |  |
| Family function |  |  |  |  | 132.9406 | <0.0001 |
| Poor | 29 (1.52) | 17 (2.18) | 5 (3.31) | 2 (4.76) |  |  |
| Fair | 73 (3.82) | 101 (12.97) | 32 (21.19) | 10 (23.81) |  |  |
| Good | 1809 (94.66) | 661 (84.85) | 114 (75.5) | 30 (71.43) |  |  |

No: EPDS score 0-9; Mild: EPDS score 10-16; Moderate: EPDS score 17-21; Severe: EPDS score 22-30.

**Table S3. Descriptive statistics for independent variables of participants during COVID-19 epidemic.**

| **Variables** | **No**  **N (%)** | **Mild**  **N (%)** | **Moderate**  **N (%)** | **Severe**  **N (%)** | ***χ^2^*** | ***P*** |
| --- | --- | --- | --- | --- | --- | --- |
| Period of COVID-19 epidemic | |  |  |  | 46.3795 | <0.0001 |
| 31 Dec. 2019-5 Jan. 2020 | 78 (4.08) | 25 (3.21) | 2 (1.32) | 1 (2.38) |  |  |
| 6 Jan.-12 Jan. | 177 (9.26) | 71 (9.11) | 14 (9.27) | 3 (7.14) |  |  |
| 13 Jan.-19 Jan. | 167 (8.74) | 61 (7.83) | 10 (6.62) | 4 (9.52) |  |  |
| 20 Jan.-26 Jan. | 103 (5.39) | 39 (5.01) | 8 (5.30) | 0 (0) |  |  |
| 27 Jan.-2 Feb. | 143 (7.48) | 73 (9.37) | 13 (8.61) | 6 (14.29) |  |  |
| 3 Feb.-9 Feb. | 134 (7.01) | 78 (10.01) | 20 (13.25) | 3 (7.14) |  |  |
| 10 Feb.-16 Feb. | 213 (11.15) | 99 (12.71) | 25 (16.56) | 11 (26.19) |  |  |
| 17 Feb.-23 Feb. | 223 (11.67) | 113 (14.51) | 22 (14.57) | 5 (11.90) |  |  |
| 24 Feb.-1 Mar. | 203 (10.62) | 93 (11.94) | 12 (7.95) | 4 (9.52) |  |  |
| 2 Mar.-8 Mar. | 193 (10.10) | 50 (6.42) | 11 (7.28) | 3 (7.14) |  |  |
| 9 Mar.-15 Mar. | 166 (8.69) | 44 (5.65) | 7 (4.64) | 1 (2.38) |  |  |
| 16 Mar.-22 Mar. | 111 (5.81) | 33 (4.24) | 7 (4.64) | 1 (2.38) |  |  |

No: EPDS score 0-9; Mild: EPDS score 10-16; Moderate: EPDS score 17-21; Severe: EPDS score 22-30.
